# High Specificity of BCL11B and GLG1 for EWSR1-FLI1 and EWSR1-ERG Positive Ewing Sarcoma

**DOI:** 10.3390/cancers12030644

**Published:** 2020-03-10

**Authors:** Martin F. Orth, Tilman L. B. Hölting, Marlene Dallmayer, Fabienne S. Wehweck, Tanja Paul, Julian Musa, Michaela C. Baldauf, Didier Surdez, Olivier Delattre, Maximilian M. L. Knott, Laura Romero-Pérez, Merve Kasan, Florencia Cidre-Aranaz, Julia S. Gerke, Shunya Ohmura, Jing Li, Aruna Marchetto, Anton G. Henssen, Özlem Özen, Shintaro Sugita, Tadashi Hasegawa, Takayuki Kanaseki, Stefanie Bertram, Uta Dirksen, Wolfgang Hartmann, Thomas Kirchner, Thomas G. P. Grünewald

**Affiliations:** 1 Max-Eder Research Group for Pediatric Sarcoma Biology, Institute of Pathology, Faculty of Medicine, LMU Munich, 80337 Munich, Germany; Martin.Orth@med.uni-muenchen.de (M.F.O.); Marlene.Dallmayer@ukmuenster.de (M.D.); Fabienne.Wehweck@med.uni-muenchen.de (F.S.W.); Tanja.Paul@med.uni-muenchen.de (T.P.); Julian.Musa@med.uni-muenchen.de (J.M.); Michaela.Baldauf@med.uni-muenchen.de (M.C.B.); Maximilian.Knott@med.uni-muenchen.de (M.M.L.K.); Laura.Romeroperez@med.uni-muenchen.de (L.R.-P.); Merve.Kasan@med.uni-muenchen.de (M.K.); Florencia.Cidre_Aranaz@med.uni-muenchen.de (F.C.-A.); Julia.Gerke@med.uni-muenchen.de (J.S.G.); Shunya.Ohmura@med.uni-muenchen.de (S.O.); Jing.Li@med.uni-muenchen.de (J.L.); Aruna.Marchetto@med.uni-muenchen.de (A.M.); 2 Department of Pediatrics, University Hospital Münster, 48149 Münster, Germany; 3 Institute of Pathology, Faculty of Medicine, LMU Munich, 80337 Munich, Germany; Thomas.Kirchner@med.uni-muenchen.de; 4 Department of General, Visceral and Transplantation Surgery, University of Heidelberg, 69120 Heidelberg, Germany; 5 INSERM Unit 830 ‘Genetics and Biology of Cancers’, Institut Curie Research Center, 75248 Paris, France; Didier.Surdez@curie.fr (D.S.); Olivier.Delattre@curie.fr (O.D.); 6 Department of Pediatric Hematology/Oncology/BMT, Charité–University Medicine Berlin, 13353 Berlin, Germany; anton.henssen@charite.de; 7 Experimental and Clinical Research Center (ECRC) of the MDC and Charité Berlin, 13125 Berlin, Germany; 8 Department of Pathology, Başkent University Hospital, Ankara 06490, Turkey; ozlemis@yahoo.com; 9 Department of Surgical Pathology, Sapporo Medical University, Sapporo 060-8556, Japan; ssugita@sapmed.ac.jp (S.S.); hasegawa@sapmed.ac.jp (T.H.); 10 Department of Pathology, Sapporo Medical University, Sapporo 060-8556, Japan; kanaseki@sapmed.ac.jp; 11 Institute of Pathology, University Hospital Essen, 45147 Essen, Germany; Stefanie.Bertram@uk-essen.de; 12 Pediatrics III, AYA Unit, West German Cancer Centre, University Hospital Essen, 45147 Essen, Germany; Uta.Dirksen@uk-essen.de; 13 German Cancer Consortium (DKTK), Partner Site Essen, 45147 Essen, Germany; 14 Division of Translational Oncology, Gerhard-Domagk-Institute for Pathology, University Hospital Münster, 48149 Münster, Germany; Wolfgang.Hartmann@ukmuenster.de; 15 German Cancer Consortium (DKTK), Partner Site Munich, 80336 Munich, Germany; 16 German Cancer Research Center (DKFZ), 69120 Heidelberg, Germany

**Keywords:** Ewing sarcoma, biomarkers, immunohistochemistry, diagnostics, BCL11B, GLG1

## Abstract

Ewing sarcoma (EwS) is an aggressive cancer displaying an undifferentiated small-round-cell histomorphology that can be easily confused with a broad spectrum of differential diagnoses. Using comparative transcriptomics and immunohistochemistry (IHC), we previously identified BCL11B and GLG1 as potential specific auxiliary IHC markers for *EWSR1-FLI1*-positive EwS. Herein, we aimed at validating the specificity of both markers in a far larger and independent cohort of EwS (including *EWSR1-ERG*-positive cases) and differential diagnoses. Furthermore, we evaluated their intra-tumoral expression heterogeneity. Thus, we stained tissue microarrays from 133 molecularly confirmed EwS cases and 320 samples from morphological mimics, as well as a series of patient-derived xenograft (PDX) models for BCL11B, GLG1, and CD99, and systematically assessed the immunoreactivity and optimal cut-offs for each marker. These analyses demonstrated that high BCL11B and/or GLG1 immunoreactivity in CD99-positive cases had a specificity of 97.5% and an accuracy of 87.4% for diagnosing EwS solely by IHC, and that the markers were expressed by *EWSR1-ERG*-positive EwS. Only little intra-tumoral heterogeneity in immunoreactivity was observed for differential diagnoses. These results indicate that BCL11B and GLG1 may help as specific auxiliary IHC markers in diagnosing EwS in conjunction with CD99, especially if confirmatory molecular diagnostics are not available.

## 1. Introduction

Ewing sarcoma (EwS) is an undifferentiated small-round-cell sarcoma driven by FET-ETS fusion oncoproteins [1]. EwS constitutes the second most common bone-associated sarcoma in children, adolescents, and young adults after osteosarcoma [1,2,3]. However, EwS also occurs in soft tissue, although less frequently and predominantly in older patients [4,5].

In more than 30% of cases, micrometastases are present at time of diagnosis [6]. For these patients, outcome is rather poor with a long-term survival rate of around 25% [6,7,8], with a slightly better survival rate for patients exhibiting exclusively lung metastases [9,10]. Nevertheless, patients with localized (i.e., non-metastatic) disease can be effectively treated with combined chemotherapy and surgery [11,12,13], resulting in a long-term survival rate of more than 70% [14,15,16]. Besides metastasis, larger tumor size also correlates negatively with patients’ overall survival [8]. Although there is an ongoing debate whether time to diagnosis is associated with metastasis and survival of individuals affected by EwS [17,18], early and robust diagnosis of EwS is important to assign patients quickly to the appropriate therapy and to avoid unnecessary delay in treatment.

From a morphological point of view, histopathological diagnosis of EwS has been notoriously difficult due to its highly undifferentiated small-round-cell phenotype being shared with a variety of differential diagnoses [19], such as neuroblastoma, rhabdomyosarcoma, lymphoblastic lymphoma, desmoplastic small round cell tumor (DSRCT), and round-cell liposarcoma [20,21,22,23,24,25].

For almost three decades, the standard immunohistochemical marker for EwS has been the membranous glycoprotein CD99 [1,26], which shows a striking sensitivity but low specificity for EwS [27,28,29,30,31]. Since fusions of the FET family of genes comprising *FUS*, *EWSR1*, and potentially *TAF15*, with members of the ETS family of transcription factors (including *FLI1*, *ERG*, *ETV1/4/6* and *FEV*) are largely pathognomonic for EwS [32,33,34], molecular identification of such fusions is the most reliable diagnostic test for EwS to date [24]. The most common fusion genes are *EWSR1-FLI1* and *EWSR1-ERG*, occurring in around 85% and 10% of cases, respectively.

However, many current standard methods used for molecular diagnosis in pathology are not infallible. For instance, PCR analyses may fail to detect all FET-ETS variants unless multiplexed PCRs covering all known variants are employed [35,36]. In addition, fluorescence in situ hybridization (FISH) approaches, commonly used to detect *EWSR1* break-apart, can miss EwS cases with *FUS-ETS* fusions [37], are often challenging in *EWSR1-ERG*-positive EwS [38], or can be misleading in morphological mimics, such as DSRCT harboring pathognomonic *EWSR1-WT1* fusions [24,39,40] or *EWSR1-NFATc2* fusion positive small-round-cell sarcomas [40,41,42].

Therefore, a progressively increasing number of institutes nowadays employ more advanced approaches as auxiliary methods to confirm or rule out the diagnosis of EwS, such as next-generation sequencing (NGS) to directly sequence the fusion transcripts [43] and/or microarray-based methylation-profiling [41,44].

However, these sophisticated molecular diagnostic tools are not available at every institution worldwide and, in some cases, not feasible due to the quantity and/or quality of the available tumor specimen. Therefore, we strived to identify highly specific auxiliary diagnostic markers that can be detected by widely available and inexpensive immunohistochemistry (IHC) [27]. Using comparative transcriptomics in EwS and 20 morphological mimics, we previously identified the proteins BCL11B (B-cell lymphoma/leukemia 11B) and GLG1 (Golgi apparatus protein 1) as potential auxiliary markers that can support the diagnosis of EwS [27]. Besides *BCL11B* and *GLG1*, out of all 19,702 genes represented on the employed microarray platform, only *ATP1A1* showed significant overexpression (log2 expression difference >2) in EwS compared to samples of the 20 morphological mimics tested [27]. Hence, BCL11B, GLG1, and ATP1A1 appeared as most promising auxiliary markers for diagnosing EwS by immunohistochemistry.

BCL11B is a transcription factor mainly involved in T cell development, which was previously identified as a direct *EWSR1-FLI1* target gene contributing to the malignant phenotype of EwS [45]. *GLG1* encodes a glycoprotein of still unknown function, even though it was first described in humans in 1990 [46]. In our discovery study (Baldauf et al. 2018) comprising 47 EwS cases and 127 samples of morphological mimics on tissue microarrays (TMAs) [27], we found that both proteins are highly overexpressed in the majority of EwS primary tumors, which is likely due to binding of *EWSR1-FLI1* to super-enhancer regions spanning their encoding genes [27]. Although analyses for ATP1A1 showed the same results, ATP1A1 did neither improve sensitivity nor specificity of the marker combination, and was not further investigated [27].

In the current study, we aimed at (I) validating the high specificity of BCL11B and GLG1 overexpression for EwS in a new, much larger, and independent cohort, (II) testing the applicability of these markers also for *EWSR1-ERG*-positive EwS, and (III) determining the degree of intra-tumoral marker heterogeneity. To this end, TMAs of 453 cases including 133 EwS cases and 320 cases from 11 differential diagnoses were constructed and stained for BCL11B, GLG1, and CD99. Immunoreactivity was assessed independently by three researchers based on a modified Remmele and Stegner scoring system [47].

Our results confirm the high specificity of BCL11B and GLG1 for EwS regardless of the underlying fusion type, enabled the refinement of cut-off values for classifying cases as ‘positive’ or ‘negative’ for the given marker, and revealed intra-tumoral heterogeneity of the proposed auxiliary markers to be negligible in differential diagnoses for EwS. These findings lead us to propose a work-flow for diagnosing EwS on the basis of IHC detection of CD99, BCL11B, and GLG1, which could be widely applied independent of the availability of molecular pathology diagnostics.

## 2. Results

### 2.1. IHC for CD99 and FISH for EWSR1 Break-Apart Are Not Fully Sufficient to Diagnose EwS

**Figure 1:**
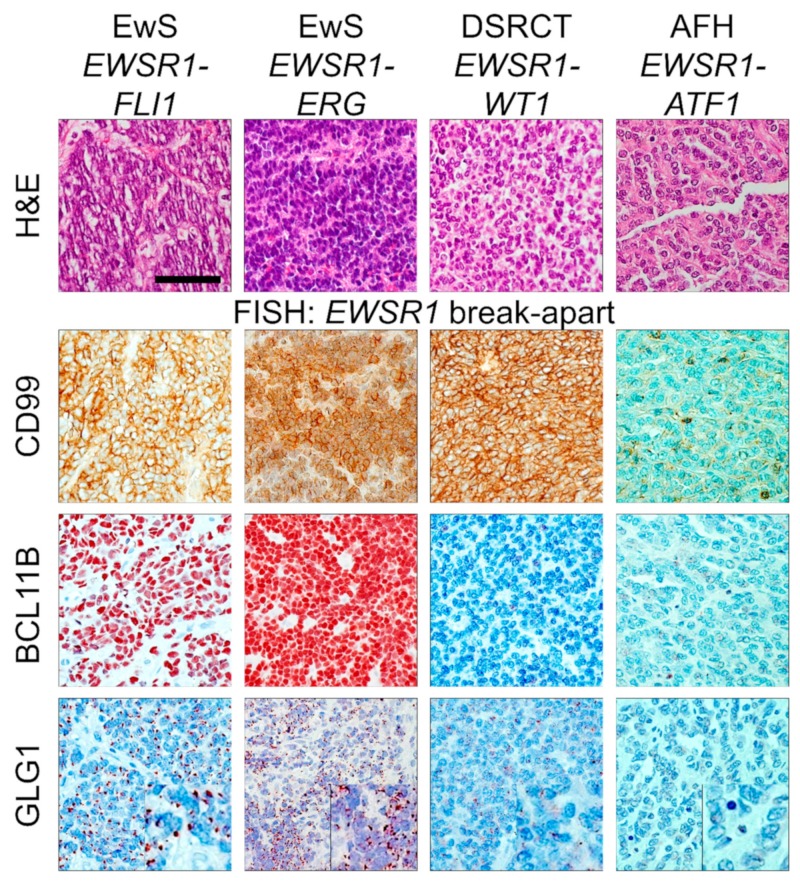
Immunohistochemistry (IHC) for CD99 and fluorescence in situ hybridization (FISH) for *EWSR1* break-apart are not sufficient to constitute the diagnosis EwS. From left to right: representative micrographs of Ewing sarcoma (EwS) positive for *EWSR1-FLI1* or *EWSR1-ERG*, desmoplastic small round cell tumor (DSRCT) positive for *EWSR1-WT1*, and an angiomatoid fibrous histiocytoma (AFH) positive for *EWSR1-ATF1*, stained with H&E, or for CD99, BCL11B, and GLG1. The scale bar applies to all micrographs and indicates 40 µm, insets for GLG1 are enlarged by factor two. In all cases, an *EWSR1* break-apart was detected by FISH.

The histological small-round-cell phenotype is shared by several cancer entities including EwS. Therefore, if such histology is present and EwS is suspected, fluorescence in situ hybridization (FISH) for detection of *EWSR1* break-apart and immunohistochemical CD99 stains are commonly performed in pathological routine diagnostics for exclusion or diagnosis of EwS [1]. Yet, *EWSR1* break-apart occurs in several morphological mimics of EwS such as DSRCT harboring a pathognomonic *EWSR1-WT1* fusion, and angiomatoid fibrous histiocytoma (AFH) commonly positive for *EWSR1-ATF1* fusions (Figure 1).

CD99 is the standard IHC marker in pathological workup of potential EwS samples [26]. However, to the best of our knowledge, no international guidelines were established on how to standardize the scoring and interpretation of CD99 immunoreactivity in EwS. In addition, CD99 is highly expressed in several other cancer types [48,49,50], and its specificity for EwS among differential diagnoses was found to be remarkably low in our previous study (17% at a cut-off of 2 [27]).

To further assess the sensitivity and specificity of CD99 immunoreactivity for EwS, we systematically scored CD99 immunoreactivity using our modified Remmele and Stegner scoring system in a series of *n* = 133 molecularly confirmed EwS tumors and *n* = 320 morphological mimics from 11 differential diagnoses (Table 1). 

In this cohort, CD99 showed high sensitivity for EwS ranging from 100% at an immunoreactive score (IRS) cut-off of >0 to 88% for an IRS cut-off of >9 (Table 2). However, the specificity of CD99 immunoreactivity proved to be rather low ranging from 2.5% to 67.5% depending on the chosen cut-off (Table 2). Even the highest CD99 IRS score of 12 was observed in some instances of morphological mimics of EwS, such as DSRCT (Figure 1). Accordingly, the high sensitivity of CD99 for EwS even at high IRS cut-offs may indicate that only strongly CD99 positive samples should be considered as potential EwS, and that samples only faintly stained for CD99 should be rather regarded as “negative”.

Collectively, the representative cases shown in Figure 1 and our systematic scoring of CD99 demonstrate that the detection of an *EWSR1* break-apart in FISH in conjunction with immunoreactivity for CD99 are not fully sufficient to robustly diagnose EwS, suggesting that more sophisticated molecular pathology techniques, such as qRT-PCR, RNA-sequencing [51,52] or comparative methylation profiling [41,44], should be employed. Alternatively, highly specific auxiliary immunohistochemical markers could be used in conjunction with CD99 to compensate for its lack of specificity. In our prior discovery study, we proposed, based on comparative transcriptomics and preliminary immunohistochemical validation, BCL11B and GLG1 as potentially useful auxiliary markers for immunohistochemical diagnosis of EwS [27]. Indeed, the high and distinct nuclear (BCL11B) or perinuclear (GLG1) expression of both markers was very helpful to establish the diagnosis of EwS in the two representative EwS cases shown in Figure 1, which were molecularly verified by next-generation sequencing (NGS; Archer FusionPlex Sarcoma panel).

### 2.2. High Immunoreactivity for BCL11B and/or GLG1 Is Specific for EwS

To assess the diagnostic value of the proposed auxiliary markers BCL11B and GLG1, all 453 cases of our new cohort (Table 1), were semi-quantitatively scored for their immunoreactivity using a modified Remmele and Stegner [47] scoring system (see Methods).

**Figure 2:**
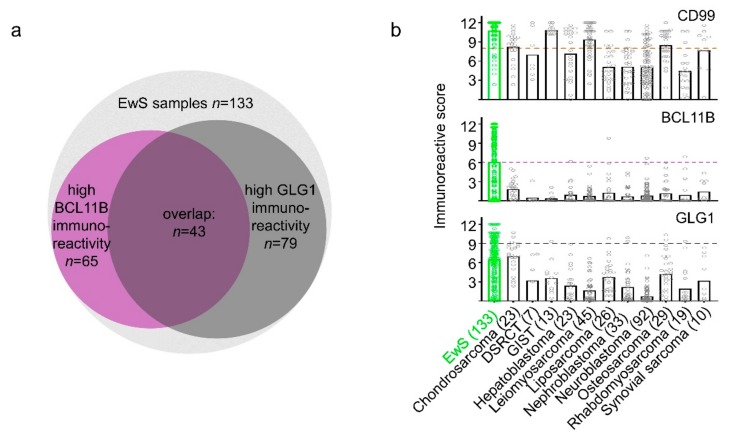
High immunoreactivity for BCL11B and GLG1 is diagnostic for EwS in CD99 positive samples. (**a**) Venn diagram representing all EwS samples in the TMA cohort of this study, the BCL11B and GLG1 high-expressing samples (at least 60% tumor cells stained, at least intermediate intensity) and their overlap. (**b**) Dot-plots indicating IRS values for CD99, BCL11B, and GLG1 in EwS and 11 differential diagnoses. The number of samples per entity is given in brackets. Bars indicate mean IRS; dashed lines indicate optimal cut-offs.

We observed that BCL11B and GLG1 were highly expressed in EwS. In fact, 75.9% of EwS cases (101/133) were highly reactive for at least one marker, co-expression of both was detected in 32.3% of EwS cases (43/133) (Figure 2a,b); 48.9% of cases (65/133) exhibited high immunoreactivity for BCL11B and 59.4% (79/133) for GLG1. High immunoreactivity was set as 60% of EwS cells per sample stained with at least intermediate intensity (IRS ≥6). 

As CD99 proved to be highly sensitive for EwS in our cohort, we considered only those samples as potential EwS with a CD99 IRS of at least 2 (100% sensitivity for EwS; Figure 2b). To define the diagnostically most informative combination of BCL11B and GLG1 immunoreactivity for EwS (only one marker positive/ at least one positive/ both positive; each marker with all different mathematically possible IRS cut-offs), we performed automated sensitivity and specificity calculations. To this end, we focused on combinations with the highest specificity for EwS, because lack of specificity is a major issue of routinely applied diagnostic tools in EwS.

After filtering out all marker combinations with a specificity for EwS <95%, we selected the marker combination with the highest sensitivity. Through this process, we noted the positivity of at least one marker with IRS cut-offs >6 and >9 for BCL11B and GLG1, respectively, to be highly specific (95.6%) but moderately sensitive (64.7%) for EwS (Figure 2b). Interestingly, CD99 with an IRS cut-off >2 did not raise the specificity of this marker combination. Only at an IRS cut-off >8 for CD99, the specificity for immunohistochemical diagnosis of EwS could be slightly increased to 97.5%, while the sensitivity slightly dropped to 63.2%.

To test, whether this marker specificity is not limited to the most common fusion type in EwS (*EWSR1-FLI1*), open slides of six molecularly confirmed *EWSR1-ERG*-positive EwS cases were stained for BCL11B and GLG1. Strikingly, BCL11B exhibited in all cases the highest possible IRS of 12 (for example see Figure 1), while the IRS for GLG1 ranged from 0–8.

We previously showed that *BCL11B* and *GLG1* mRNA expression is upregulated by *EWSR1-FLI1* in EwS cell lines harboring this specific fusion type [27]. To test whether the marker transcripts can be upregulated also by *EWSR1-ERG*, we assessed their expression by qRT-PCR in three *EWSR1-ERG*-positive EwS cell lines with/without RNA interference-mediated *EWSR1-ERG* silencing. As shown in Supplementary Figure S1a, knockdown of *EWSR1-ERG* was accompanied by downregulation of *BCL11B* and *GLG1* expression in all three cell lines tested, suggesting that these markers are also upregulated by *EWSR1-ERG* in EwS harboring this fusion (around 10% of cases). In conjunction with the scoring results of the six molecularly confirmed *EWSR1-ERG*-positive EwS cases, these experimental results further suggest that high expression of both marker proteins in primary EwS is not restricted to *EWSR1-FLI1*-positive tumors.

Since patient-derived xenografts (PDX) are increasingly used as preclinical models to study EwS biology [48], we tested the expression pattern of BCL11B and GLG1 in PDX from EwS and non-EwS patients (18 PDX models originating from 15 EwS patients; 52 PDX models originating from 49 non-EwS patients). As expected, expression of both markers was higher for PDX from EwS patients as compared to the non-EwS PDX (Supplementary Figure S1b). Interestingly, the tested series of PDX models included five samples from so-called ‘Ewing-like’ sarcomas (3 *CIC-DUX4*-positive sarcomas and 2 *BCOR*-rearranged sarcomas), which did not exhibit any BCL11B and GLG1 immunoreactivity. These findings in PDX models further support the high specificity of BCL11B and GLG1 for EwS.

### 2.3. BCL11B and GLG1 Exhibit Low Intra-Tumoral Heterogeneity in Differential Diagnoses for EwS

Tumor material is often limited in pathological workup (e.g., in needle/punch biopsies). In the context of histological tumor evaluation, limited material can lead to substantial confusion in case of high intra-tumoral heterogeneity of morphology and diagnostic marker expression. Therefore, the heterogeneity of scoring for CD99, BCL11B, and GLG1 in different cores per sample, originating from different regions of a given tumor block, was assessed. For non-EwS, 248 samples with three cores per marker were available in our TMA-cohort. Samples were classified as high or low for the different markers applying the thresholds defined above (Figure 2b).

**Figure 3:**
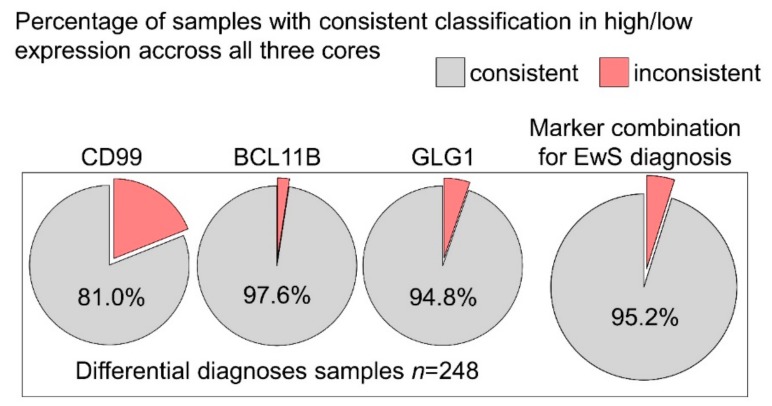
BCL11B and GLG1 show low intra-tumoral expression heterogeneity in differential diagnosis for EwS. Pie charts indicating non-EwS samples with three cores evaluated in each staining (*n* = 248) that were classified as high- or low-expressing the respective marker consistently (grey) or inconsistently (light red) across all three cores.

For CD99, 81% of non-EwS tumor samples were classified consistently across all three cores per sample, for BCL11B 97.6%, and for GLG1 and 94.8% (Figure 3), indicating only little intra-tumoral heterogeneity in EwS differential diagnoses. When combining the three markers, in 95.2% of all non-EwS samples all three cores were classified consistently as EwS or non-EwS. Hence, even when only little material of non-EwS cases is available, misclassification as EwS because of marker heterogeneity is highly unlikely, and the specificity of the markers is not affected. The observed intra-tumoral heterogeneity in differential diagnosis for EwS can therefore be regarded as rather negligible.

In our TMA-cohort, most EwS samples were represented by two cores. For *n* = 96 EwS cases two cores were present in each staining. In these 96 EwS cases, we found consistent classification as marker-positive or -negative in 81.1%–87.4% (Supplementary Figure S2). 

In sum, these results suggest that the combination of CD99 as a screening marker and the highly specific auxiliary markers BCL11B and GLG1 may be applicable even in cases where only scarce material is available.

### 2.4. Proposed Work-Flow for Immunohistochemical Assessment of EwS

Given the above-mentioned results and in support of the findings of Baldauf et al. 2018 [27], we propose the following work-flow for immunohistochemical assessment of EwS: First, CD99 staining should be performed. If the IRS is equal or lower than 8, the suspected diagnosis should be reconsidered. In cases with a CD99 IRS greater than 8 (meaning strong membranous staining intensity in at least 60% of tumor cells), the diagnosis of EwS should be either confirmed by molecular pathology approaches that can reliably detect *FET-ETS* fusions such as qRT-PCR or RNA-seq, or by immunohistochemical staining for the auxiliary markers BCL11B and GLG1.

**Figure 4:**
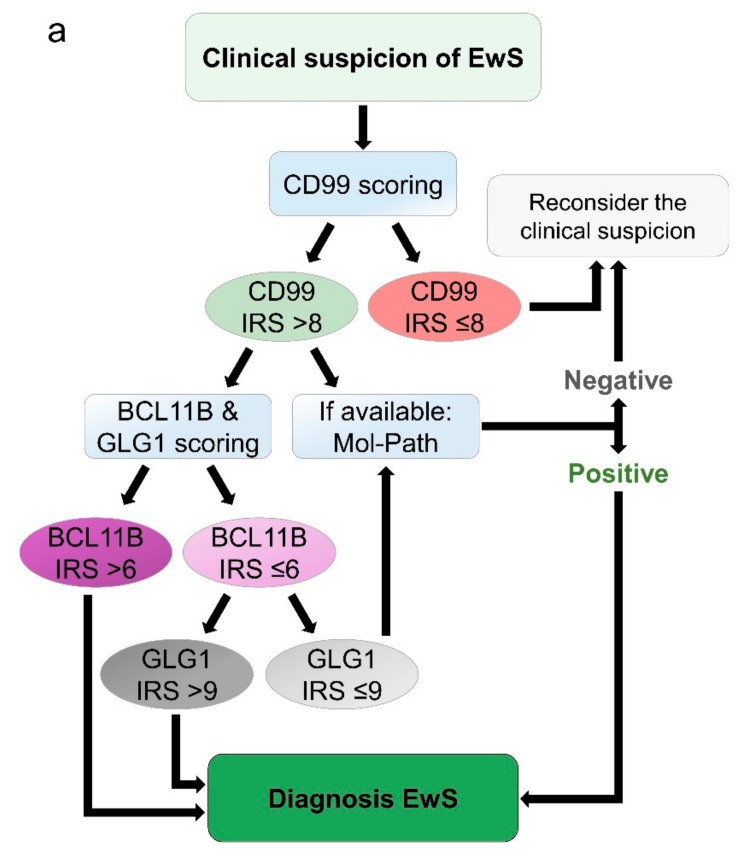

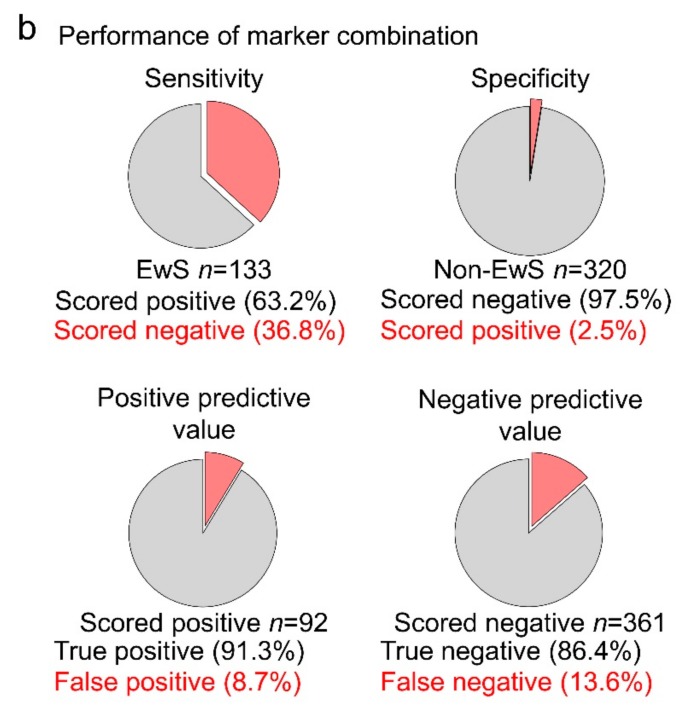
Proposed work-flow for immunohistochemical diagnosis of EwS. (**a**) Scheme of the proposed work-flow for immunohistochemical diagnosis of EwS; (**b**) pie charts indicating the performance of the proposed diagnostic procedure in the cohort of 133 EwS and 320 samples of 11 differential diagnoses.

The diagnosis of EwS can be confidently established in case of high BCL11B immunoreactivity (IRS >6; i.e., moderate nuclear staining intensity in at least 80% of tumor cells, or strong nuclear staining intensity in at least 60% of tumor cells) and/or high GLG1 immunoreactivity (IRS >9; i.e., strong perinuclear staining in at least 80% of tumor cells). If both markers show immunoreactivity scores below the respective cut-off, the diagnosis of EwS should be molecularly confirmed (Figure 4a). Applying this algorithm to our cohort of 133 EwS cases and 320 cases from differential diagnoses yields a positive predictive value of 91.3% and a negative predictive value of 86.4% (Figure 4b)

## 3. Discussion

Robust diagnostics are essential to quickly and correctly assign EwS patients to the appropriate therapy. After initial clinical presentation and imaging, the diagnostic workup of potential EwS patients is performed by surgical pathologists. As EwS belongs to the group of undifferentiated small-round-cell sarcomas [19], its histological phenotype is shared by several differential diagnoses [53].

In histopathological examination, evaluation of H&E-stained slides is routinely followed by CD99 immunostaining [26], which is commonly positive in the vast majority of EwS cases [26,54]. In accordance, the CD99 IRS for 91% (121/133) of EwS in our TMA cohort was >8.

However, even strong membranous CD99 immunoreactivity is not specific for EwS [28,55]. Indeed, 40% of non-EwS cases examined in the current study (128/320 samples, representing 11 distinct tumor entities) exhibited a strong CD99 immunostaining (IRS >8). Accordingly, in most institutions, the diagnosis of EwS is further validated by molecular pathology techniques, most commonly FISH to detect break-apart events in the *EWSR1* gene [24]. Although a positive result is highly sensitive for EwS, some morphological mimics can exhibit break-apart events in the *EWSR1* gene (e.g., DSRCT, AFH, and EWSR1-NFATc2-positive sarcomas) [39,40,41].

Another molecular technique is multiplex qRT-PCR to amplify pathognomonic *FET-ETS* fusion transcripts. However, due to the large variety of existing *FET-ETS* fusions in EwS [56], many of the currently used multiplex PCR sets likely do not cover all possible *FET-ETS* fusions. As a consequence, many institutions increasingly employ next-generation RNA-sequencing technologies to overcome these limitations. Yet, these techniques are not available or feasible in all cases, which is why a straight-forward immunohistochemical approach is still desirable.

Hence, many groups have evaluated several potential diagnostic markers such as FLI1, NKX2-2, and PAX7 that could be used instead of the highly unspecific marker CD99 [40,57,58]. However, to the best of our knowledge, none of these proposed IHC markers completely fulfills the requirements of providing very high sensitivity and specificity for EwS at the same time. While FLI1 is unable to confirm EwS cases with *FET–non-FLI1* translocations [33,59], NKX2-2 and PAX7 lack specificity [60] and can be even strongly expressed in close morphological mimics such as *EWSR1-NFATc2*-positive sarcomas [40,57,58]. Besides, also Caveolin-1 [54,61], Cyclin-D1 [62], and NR0B1 [63] have been proposed as potential (auxiliary) IHC markers for EwS. While Cyclin-D1 and NR0B1 showed high sensitivity for EwS, they were reported as being not fully specific, which is why it was proposed to use them in an IHC marker panel [62,64]. Caveolin-1 is another highly sensitive IHC marker for EwS [54] but its specificity has—to the best of our knowledge—not been systematically assessed yet. The fact that *Caveolin-1*, *Cyclin-D1*, *NKX2-2*, *NR0B1* and *PAX7* are known FET-ETS target genes [57,61,63,65,66] implies that not every FET-ETS target gene may serve as a highly specific auxiliary marker, since they may be also expressed at high levels in some morphological mimics.

To this end, we set out to identify highly specific auxiliary markers that can compensate the lack of specificity of CD99. By comparative transcriptome profiling of EwS cases and hundreds of samples from 20 morphological mimics, we previously identified three highly specific marker candidates, namely *ATP1A1*, *BCL11B*, and *GLG1* [27]. Using IHC in a series of 47 EwS and 127 non-EwS tumors from 11 distinct entities, we found that high BCL11B and/or high GLG1 expression in CD99-positive cases may be diagnostic for EwS, while ATP1A1 did neither raise the specificity nor sensitivity of this marker combination further [27]. However, even broader transcriptome analyses covering more than the investigated 19,702 genes and/or comparative proteomics analyses could yield additional auxiliary markers that are currently unknown.

In the current study, we confirmed the high specificity of BCL11B and GLG1 for EwS in a far larger and independent cohort of 133 EwS and 320 non-EwS from 11 differential diagnoses, including 118 samples from close morphological mimics such as DSRCT, neuroblastoma, and rhabdomyosarcoma. This high number of samples enabled a refinement of the IRS cut-offs for both markers (BCL11B >6, GLG1 >9; compared to previously proposed cut-offs >9 for both markers). In conjunction with the highly sensitive marker CD99, our approach yielded a specificity of 97.5% and an accuracy of 87.4% for diagnosing EwS solely by IHC. Also, EwS cases with *EWSR1-ERG* fusions exhibited high marker immunoreactivity. 

However, it should be noted that in our cohort, 36.8% of EwS cases could not be confirmed by this marker combination. If, in these cases, there is strong clinical suspicion of EwS, additional molecular approaches are mandatory for diagnosis. Additionally, in the current study, we used specific polyclonal antibodies against BCL11B and GLG1 (see methods). To further improve the accuracy of our diagnostic IHC approach and to reduce the risk of batch effects across different polyclonal antibodies, we recommend to systematically develop and improve monoclonal antibodies against both antigens that can be employed for IHC.

As tumor material is often limited, heterogeneously expressed markers can lead to misdiagnoses depending on the tumor fraction captured in a given sample. Hence, homogeneous expression of potential markers within tumors is desirable. For non-EwS cases, the proposed marker combination of CD99, BCL11B, and GLG1 yielded a consistent classification over all 3 cores taken from different areas of each tumor sample in 95.2%. These findings suggest that the specificity of our approach is likely not compromised even when only little tumor material is available for diagnostics. Marker homogeneity in EwS was only moderate, possibly explaining the observed moderate sensitivity of our markers in our TMA cohort (representing each EwS sample with two 1 mm cores, in other words 1.6 mm^2^ tumor material). Potentially, the sensitivity might be even higher when more tumor material is available. 

It should be noted that both auxiliary markers need to be carefully scored according to a modified Remmele and Stegner IRS. That is, both markers do not exhibit an ‘all-or-nothing’ expression pattern and rather display substantial expression variability in EwS and non-EwS cases. Thus, the actual immunoreactivity needs to be compared to visual analogous scales (see Baldauf et al. 2018 [27]), especially by pathologists with little experience with these markers. Yet, since most non-EwS cases do not exhibit any or only very weak positivity for both markers, and EwS exhibit very strong positivity, we believe that our auxiliary markers could easily be implemented on routine IHC workup of suspected EwS cases.

## 4. Materials and Methods 

### 4.1. Human Samples and Ethics Approval

The study was conducted in accordance with the Declaration of Helsinki. Formalin-fixed paraffin-embedded (FFPE) human tissue samples of EwS and differential diagnoses were retrieved from the archives of the Institute of Pathology of the LMU Munich (years 2009 to 2019) and collaborating institutions with approval of the ethics committee of the LMU Munich University Hospital (307-16 UE). Patient derived xenografts (PDX) were provided by the Childhood Solid Tumor Network [67], D. Surdez and O. Delattre (Paris, France), and A. Henssen (Berlin, Germany) with approval of the corresponding institutional review boards.

### 4.2. Construction of Tissue Microarrays (TMA) and Immunohistochemistry (IHC)

A total of 348 FFPE primary tissue samples and FFPE PDX samples from 64 pediatric sarcomas were collected. Only samples that could be diagnosed unambiguously using current state-of-the-art diagnostics for the respective tumor entity, were further processed. For each tumor, all FFPE blocks were examined in H&E stained slides and the tumor block containing the largest amount of vital tumor tissue was selected for TMA construction (three cores per tumor block, each with a diameter of 1 mm, and internal controls). Additionally, TMAs with 144 EwS samples and at least two cores per sample were obtained from W. Hartmann and U. Dirksen (Münster and Essen, Germany). The composition of the final cohort comprising *n* = 453 samples is given in Table 1.

For IHC, newly cut 4 μm sections of TMAs or available open slides for *n* = 6 *EWSR1-ERG* positive EwS samples, *n* = 1 *EWSR1-ATF1* positive angiomatoid fibrous histiocytoma (AFH), and *n* = 12 PDX models were used. Antigen retrieval was performed with microwave treatment, 2 × 15 min at 750 W using the antigen retrieval AR-10 solution (HK057-5K, DCS Innovative, Hamburg, Germany) for GLG1 and the Dako Target Retrieval Solution (S1699, Agilent, Santa Clara, CA, USA) for BCL11B. After rinsing the slides with pH 7.5 TRIS buffer (0.05 M; Sigma, Taufkirchen, Germany), endogenous peroxidase was blocked by 7.5% aqueous H_2_O_2_ solution at room temperature and blocking serum from the corresponding kits for 20 min.

Slides were then incubated for 60 min with primary rabbit-anti-human antibodies anti-BCL11B (1:1000 dilution; ab70453, Abcam, Cambridge, UK), or anti-GLG1 (1:250 dilution; HPA010815, Atlas Antibodies, Bromma, Sweden). Then slides were incubated with a secondary anti-rabbit IgG antibody (ImmPress Reagent Kit, peroxidase-conjugated) followed by target detection using AEC plus chromogen for 10 min (K3461, Agilent).

For IHC staining of CD99, 4 μm sections were incubated for 32 min with a monoclonal mouse-anti-human antibody (1:40 dilution; 12E7, Agilent) using the ultraView detection kit in a VENTANA BenchMark system (Roche, Basel, Switzerland).

### 4.3. Evaluation of Immunoreactivity and Automated Cut-Off Finding

Immunoreactivity of all TMAs and open slides for CD99, BCL11B, and GLG1 was semi-quantitatively scored by three independent physician-scientists in analogy to scoring of hormone receptors with the immunoreactive score (IRS) established by Remmele and Stegner [47] with slight modifications of the grading of the percentage of positive tumor cells. First, the percentage of stained tumor cells was determined as the average of at least three high-power fields (40×) and rated on a scale of 0–4 (grade 0 = 0%–19%, grade 1 = 20%–39%, grade 2 = 40%–59%, grade 3 = 60%–79%, and grade 4 = 80%–100%). Second, for samples with marker immunoreactivity, the most representative intensity was determined (grade 1 = low, grade 2 = moderate, and grade 3 = strong staining intensity). The product of these two grades defined the final IRS. After filtering out samples without representative tumor material on TMAs, the few samples with high inter-observer scoring deviation were revised and a consensus was built. High inter-observer scoring deviation was assumed if the sum of the absolute differences between the score of each observer and the mean IRS was at least as high as the mean IRS. Automated cut-off finding and combination testing were performed with an in-house VBA script implemented in Microsoft Excel (2016, 32-bit) as described in our discovery study [27].

### 4.4. Evaluation of Heterogeneity of Intra-Tumoral Marker Expression

To test whether the markers investigated in this study were heterogeneously expressed in a given differential diagnosis for EwS, we selected all tumor samples for which representative material was present in all three cores of the respective TMA for CD99, BCL11B, and GLG1 staining. In a next step, we evaluated for each core whether the IRS values for CD99, BCL11B, and GLG1 exceeded the predefined cut-offs (>8, >6, >9, respectively, see Results section) to classify each core as either marker high/positive or low/negative. Then, we assessed whether the results were consistent across all three cores per tumor sample. As most EwS cases were represented by two cores per sample, the evaluation of marker expression heterogeneity for EwS was performed separately on samples with two cores present in each staining.

### 4.5. RNA Extraction, Reverse Transcription, and Quantitative Real-Time PCR (qRT-PCR)

To test for potential transcriptional regulation of *BCL11B* and *GLG1* by *EWSR1-ERG*, we employed clones of three *EWSR1-ERG* positive EwS cell lines (CHLA25, EW3, and TC106) harboring a doxycycline inducible shRNA mediated *EWSR1-ERG* knockdown system. Wild-type CHLA25 and TC106 cells were obtained from the Children’s Oncology Group (COG), and EW3 cells were kindly provided by O. Delattre. Cell line authenticity was confirmed by STR-profiling and mycoplasma contamination was ruled out by nested PCR. Total RNA was extracted from these EwS cell lines 96 h after induction of *EWSR1-ERG* knockdown using the NucleoSpin RNA kit (Macherey-Nagel, Düren, Germany). RNA was reversely transcribed using the High-Capacity cDNA Reverse Transcription Kit (Applied Biosystems, Foster City, CA, USA). qRT-PCRs were performed with 1:10 diluted cDNA and 0.5 µM forward and reverse primers in SYBR Green PCR Master Mix (Applied Biosystems). Oligonucleotides were purchased from MWG Eurofins Genomics (Ebersberg, Germany). The following primer sequences were used:

*BCL11B* forward: 5′-ATGCCAGAATAGATGCCGG-3′;
*BCL11B* reverse: 5′-CTCTATCTCCAGACCCTCGTC-3′;
*GLG1* forward: 5′-GTGGAGTGTAGAGATATAGTTGGC-3′;
*GLG1* reverse: 5′-ATCAGGTCCCCAGAGTCTATC-3′;
*RPLP0* forward: 5′-GAAACTCTGCATTCTCGCTTC-3′;
*RPLP0* reverse: 5′-GGTGTAATCCGTCTCCACAG-3′.

Reactions were run on a BioRad CFX Connect instrument (Bio-Rad, Munich, Germany) with the following thermal conditions: heat activation (95 °C, 2 min), DNA denaturation (95 °C, 10 s), annealing and elongation (60 °C, 30 s), 49 repeat cycles, final denaturation (95°C, 30 s). *RPLP0* was used as housekeeping gene [68].

## 5. Conclusions

We anticipate that the here proposed diagnostic procedure (Figure 4a) will provide robust diagnosis in a large fraction of EwS cases even without any additional molecular diagnostic techniques, which may be of great benefit for patients, especially in settings in which molecular diagnostic approaches are not available or feasible.

## Figures and Tables

**Table 1:** Composition of the tissue microarrays (TMAs). EwS, Ewing sarcoma; DSRCT, desmoplastic small round cell tumor; GIST, gastrointestinal stromal tumor; dediff, dedifferentiated.

Tumor Entity	*n*
EwS	133
Chondrosarcoma	23
DSRCT	7
GIST	13
Hepatoblastoma	23
Leiomyosarcoma	45
Liposarcoma (dediff./myxoid)	26 (17/9)
Nephroblastoma	33
Neuroblastoma	92
Osteosarcoma	29
Rhabdomyosarcoma	19
Synovial sarcoma	10
total	453

**Table 2:** Sensitivity and specificity of CD99 immunoreactivity in EwS. IRS, immunoreactive score.

Cut-Off (CD99 IRS > Indicated Value)	Sensitivity (%)	Specificity (%)
0	100.0	2.5
1	100.0	6.6
2	100.0	10.0
3	99.2	18.1
4	98.5	26.9
6	95.5	43.8
8	91.0	60.0
9	88.0	67.5

## Supplementary Materials

The following are available online at https://www.mdpi.com/2072-6694/12/3/644/s1, Figure S1: *EWSR-ERG* knockdown leads to decrease of BCL11B and GLG1 expression in EwS cell line models and PDX EwS models exhibit high immunoreactivity for BCL11B and GLG1; Figure S2: BCL11B and GLG1 show moderate intra-tumoral staining heterogeneity in EwS.
