# Supplementary material for: High Specificity of BCL11B and GLG1 for EWSR1-FLI1 and EWSR1-ERG Positive Ewing Sarcoma
Source: Cancers (Basel). 2020 Mar 10;12(3):644. doi: 10.3390/cancers12030644 (PMC7139395; doi:10.3390/cancers12030644)
Supplement: Supplementary file 1 [file cancers-12-00644-s001.zip › Suppl.Fig.S1_Orth_etal.pdf]

Supplementary Figure S1 Orth *et al.*

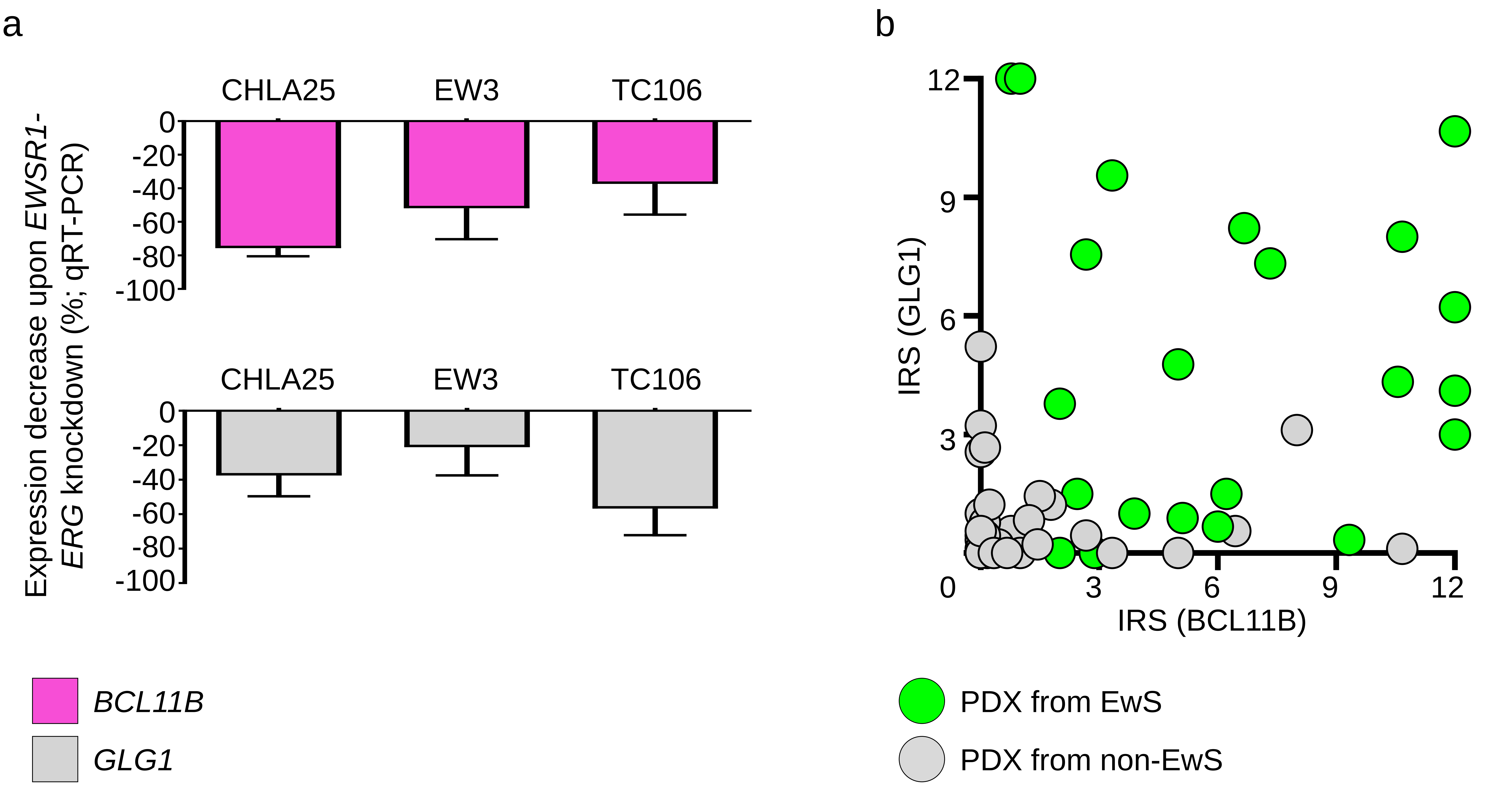

Supplementary Figure S1: *EWSR-ERG* knockdown leads to decrease of *BCL11B* and *GLG1* expression in EwS cell line models and PDX EwS models exhibit high immunoreactivity for BCL11B and GLG1. (**a**) Bar plots indicating expression decrease of *BCL11B* (pink) and *GLG1* (grey) upon knockdown of the fusion oncogene in three *EWSR1-ERG* positive EwS cell lines, qRT-PCR results of *n*=3 experiments, error bars indicate standard deviation. (**b**) Diagram indicating BCL11B and GLG1 immunoreactivity for PDX from EwS (green) and other pediatric sarcomas (grey).
