# Supplementary material for: High Specificity of BCL11B and GLG1 for EWSR1-FLI1 and EWSR1-ERG Positive Ewing Sarcoma
Source: Cancers (Basel). 2020 Mar 10;12(3):644. doi: 10.3390/cancers12030644 (PMC7139395; doi:10.3390/cancers12030644)
Supplement: Supplementary file 1 [file cancers-12-00644-s001.zip › Suppl.Fig.S2_Orth_etal.pdf]

Supplementary Figure S2 Orth *et al.*

Percentage of samples with consistent classification in high/low expression accross both cores

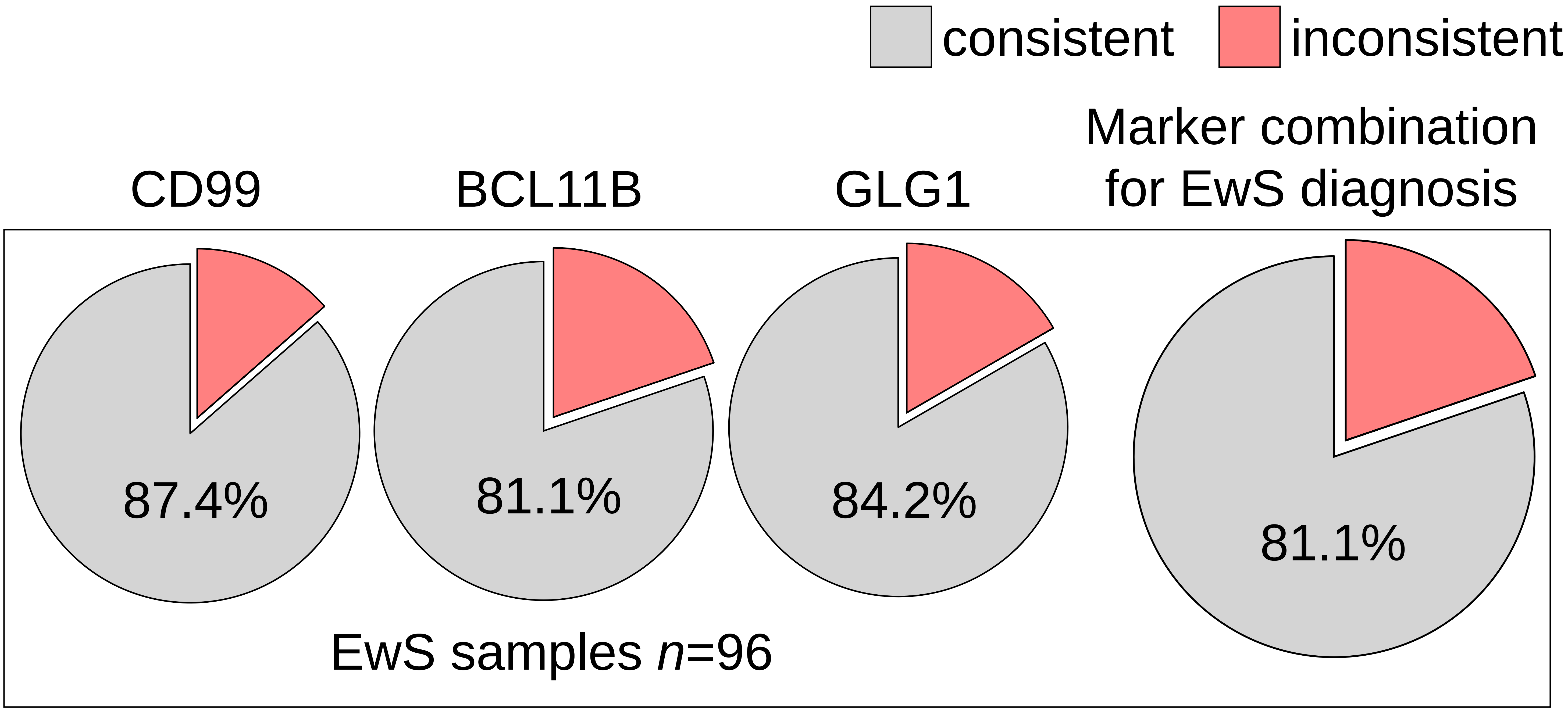

Supplementary Figure S2: BCL11B and GLG1 show moderate intra-tumoral staining heterogeneity in EwS. Pie charts indicating EwS samples with two cores evaluated in each staining (*n*=96) that were classified as high or low expressing the respective marker consistently (grey) or inconsistently (light red) across both cores.
